# Factors Associated With Health Perception and Psychological Well-Being Among Third-Age University Students: Cross-Sectional Study on the Role of Cyberchondria

**DOI:** 10.2196/83371

**Published:** 2026-08-21

**Authors:** Semra Gündoğdu, Özlem Özgür, Asli Kilavuz, İsmail Tufan

**Affiliations:** 1 Department of Internal Medicine Nursing, Faculty of Nursing, Akdeniz University, Antalya, Türkiye; 2 Department Of Gerontology, Faculty Of Health Sciences, Akdeniz University, Antalya, Türkiye; 3 Department of Internal Medicine, Division of Geriatrics, Faculty of Medicine, Ege University, 5th floor, Bornova, Izmir, 35100, Türkiye, 90 5323536570, 90 2323437876

**Keywords:** cyberchondria, third-age university, health perception, psychological well-being, older adults

## Abstract

**Background:**

With Türkiye’s aging population and widespread internet usage, the excessive seeking of health information through online platforms, formally identified as cyberchondria, has emerged as a concern affecting older individuals’ health perception and psychological well-being.

**Objective:**

This study aimed to evaluate the association of cyberchondria with health perceptions and psychological well-being among older participants in a third-age university program.

**Methods:**

This cross-sectional study included 352 participants aged ≥60 (mean 67.4, SD 4.7) years from Tazelenme University, Antalya, Türkiye, and was conducted between November and December 2024. Data were collected using the Cyberchondria Severity Scale Short Form (CSS-12), Individual Health Perception Scale, and Psychological Well-Being Scale for Older People (PWBS-OP). Statistical analyses included correlation and multiple regression analyses.

**Results:**

The mean age of the 352 participants was 67.4 (SD 4.7, range 60-87) years, 38.6% (n=136) were first-year students, 69.6% (n=245) were men, 58.8% (n=207) were married, 48.9% (n=172) were university graduates or above, and 65.1% (n=229) had chronic diseases. Significant negative correlations were found between CSS-12 distress, compulsion, and total scores with both Individual Health Perception Scale and PWBS-OP scores (*P*<.05). According to the multiple linear regression analysis, the presence of chronic disease was the only significant positive predictor of higher health perception levels (*B*=2.16, 95% CI 1.721-3.593; *P*=.003), whereas factors such as age, gender, education level, and CSS-12 total scores did not demonstrate a statistically significant impact (*P*>.05). Psychological well-being (PWBS-OP) was significantly and positively predicted by age (*B*=0.22, 95% CI 0.037-0.399; *P*=.02), marital status (*B*=1.67, 95% CI 1.064-2.716; *P*=.002), economic status (*B*=2.10, 95% CI 1.729-3.464; *P*=.003), and the thought of having an undiagnosed disease (*B*=3.38, 95% CI 1.038-5.830; *P*=.007), whereas it was significantly and negatively predicted by medical examinations in the past year (*B*=−1.29, 95% CI −2.093 to 0.485; *P*=.002), undergoing examinations without a physician’s recommendation (*B*=−2.04, 95% CI −3.794 to −0.290; *P*=.02), searching for health-related topics on the internet (*B*=−1.26, 95% CI −2.152 to −0.360; *P*=.006), and CSS-12 total scores (*B*=−0.13, 95% CI −0.261 to −0.060; *P*=.04).

**Conclusions:**

High cyberchondria levels significantly impair older adults’ psychological well-being and health perception. While demographic factors positively influence health perception, excessive internet-based health seeking deteriorates psychological well-being. Digital health literacy programs, professional online health counseling, and psychological support should be recommended to target cyberchondria risks in older populations.

## Introduction

In alignment with global demographic shifts, Türkiye is experiencing a significant increase in its older population. In 2024, the older population was 10.6% of Türkiye’s total population and is expected to increase to 13.5% by 2030 [1]. Parallel to this demographic transition, technology has become an indispensable component of daily activities, education, and health care [2]. Modern society necessitates that older adults engage with new information and communication technologies to maintain independence, safety, and social connectivity. Research shows that the percentage of individuals aged ≥65 years who search for health information online rose from 14.5% in 2011 to 43.6% in 2020 [3]. Consequently, internet usage among older adults in Türkiye reached 46.9% in 2024, with health-related information seeking emerging as one of the most frequent online activities [1]. While these digital tools empower older adults in health management and decision-making, the unregulated nature of online information has introduced a distinct challenge: the phenomenon of cyberchondria.

Cyberchondria is defined as the excessive and compulsive seeking of medical information online, a behavior typically driven by and resulting in heightened health anxiety [4-6].

The dissemination of misleading or incomplete information, combined with the internet’s capacity to reinforce preexisting fears, creates a reinforcing cycle in which search attempts intended to provide relief instead exacerbate distress [7]. This information pollution directly undermines efforts to achieve “complete well-being,” which the World Health Organization (WHO) defines not merely as the absence of disease, but as a state of total physical, mental, and social wellness [8]. Within this holistic framework, cyberchondria poses a significant threat to an individual’s subjective evaluation of their own health and their overall mental equilibrium.

The subjective assessment of an individual’s physical and mental state is conceptualized as “health perception.” Health perception involves a complex blend of feelings and thoughts that may not always align with objective, clinically determined health status [9].

In the context of cyberchondria, intensive exposure to disease symptoms online can lead individuals to select and adapt these symptoms to their own self-image, potentially deteriorating their health perception even in the absence of clinical illness.

Beyond perception, this behavior also impacts “psychological well-being,” a multidimensional construct encompassing self-acceptance, environmental mastery, and autonomy. When older adults encounter inconsistent or frightening health information, it can diminish their sense of environmental mastery and autonomy, thereby reducing their overall quality of life [7].

To mitigate these risks and promote active aging, “lifelong learning” programs have become vital interventions. In Türkiye, the 60+ Tazelenme University model provides a platform for education and social engagement that has been shown to improve life satisfaction and self-health evaluations among participants. However, while these students are encouraged to use technology for healthy aging, their vulnerability to cyberchondria and its subsequent impact on their psychological and perceptual health remains an area requiring closer investigation.

A study also showed that older participants from third-age universities improved life satisfaction, subjective well-being, and health habits [10]. Third-age university students showed improved self-care behaviors in comparison to their nonparticipant peers and reported better self-health evaluations [11]. This reveals that education and social engagement help senior citizens with their health perception and psychological wellness. However, the amount and quality of online health information, to some extent, is disruptive, especially the cyberchondria phenomenon, which manifests as excessive and anxiety-driven searches. It is likely to exacerbate the perception and mental health of older adults.

Third-age university students are an illustrative example of the so-called “active agers,” and this group has a higher degree of motivation for self-care and social involvement than the average older adult. Nevertheless, the paradox of this greater digital involvement stems from the fact that, although this group, being engaged in education, ought to be the most psychologically protected, the existence of an “at-risk“ phenomenon, in health-related internet searching, may, for multiple reasons, increase their exposure to the internet and, as a consequence, increase health-related misinformation, health-related anxiety, and cyberchondria. As of now, the literature has completely overlooked the question of whether the environment of lifelong education has the potential to counteract the negative psychological effects of cyberchondria. For this reason, this study aimed to provide answers to the following hypotheses:

- Hypothesis 1: Higher levels of cyberchondria are correlated to lower health perceptions of older adults participating in lifelong learning programs.
- Hypothesis 2: Higher levels of cyberchondria are correlated to lower psychological well-being of those individuals.
- Hypothesis 3: Specific sociodemographic and digital usage patterns are significant predictors of health perception and well-being within this educational context.

Given this context, understanding the relationship between internet-based health seeking and the holistic well-being of older adults is essential for developing effective digital health literacy and psychological support programs. Therefore, this study aimed to evaluate the association of cyberchondria with health perceptions and psychological well-being among older participants enrolled in a third-age university program.

## Methods

### Study Design

This study is a descriptive and cross-sectional study conducted at the Tazelenme University of Antalya Campus between November and December 2024. 60+ Tazelenme University is a third-age university established as a social responsibility project that has been providing 4 years of free education since 2016 with the principle of lifelong learning for individuals aged ≥60 years.

### Study Sample

The research population comprised 643 Tazelenme University students enrolled in the program conducted within the scope of Akdeniz University Center for Applied Research on Aging Studies (1st grade: n=215, 33.4%; 2nd grade: n=184, 28.6%; 3rd grade: n=124, 19.3%; and 4th grade: n=120, 18.7% students) [12]. The sample size was calculated using the “Sample Size for % Frequency in a Population” module in the OpenEpi program [13] for a cross-sectional study. The population size was taken as 643, the expected frequency or proportion was determined as 50% because no prior estimate was available, the confidence level was set at 99%, the absolute margin of error was set at 5%, and the design effect was set at 1.0 under the assumption of simple random sampling. Under these assumptions, the minimum sample size required was calculated as 327. Inclusion criteria for the research were being aged ≥60 years, being a Tazelenme University student, and volunteering to participate in the research. Noninclusion criteria for the study included being aged <60 years, not being officially enrolled in the specified university campus, and not having internet access or a valid email address, as an online survey platform was used for data collection. Exclusion criteria included surveys containing incomplete or inaccurate data and withdrawal of consent at any point during the survey process.

### Data Collection Tools

Data were collected using a “Personal Information Form,” “Cyberchondria Severity Scale Short Form (CSS-12)” [14], Individual Health Perception Scale (IHPS) [15,16], and the Psychological Well-Being Scale for the Older People (PWBS-OP) [17].

The data were collected between November and December 2024 using an online survey form created with Google Forms. The survey form was sent to the students’ email addresses. The survey took approximately 15 minutes to complete. The research surveys were conducted in a single session.

### Personal Information Form

#### Overview

This form was created by the researchers and included questions related to demographic information, such as age, class (1, 2, 3, or 4), gender (female or male), marital status (married or single), family type (nuclear or extended), and educational status (the educational status of the participants refers to the highest level of formal education completed before joining the third-age university program). As 60+ Tazelenme University is a social responsibility project based on the principle of lifelong learning, it accepts students from all educational backgrounds, provided they meet the age requirement (primary school, secondary school, high school, university, or higher), marital status (married, divorced, widowed, or single), socioeconomic status (income less than expenditure, income equals expenditure, and more income than expenditure), presence of health insurance (yes or no), smoking status (nonsmoker, ex-smoker, or current smoker), living arrangement (with spouse, alone, spouse and children, or children), number of physician visits in the past year (never, 1‐3 times, 4‐6 times, 7‐9 times, or ≥10 times), daily internet use duration (1‐2 h, 3-4 h, 5-6 h, or >6 h), the source for accessing health information (health care professionals, TV or radio, printed press, internet, family members, or friends).

#### CSS-12

The scale was developed by McElroy et al [14]. The Turkish validity and reliability study of the scale was conducted by Tuğtekin and Tuğtekin, reporting a Cronbach α coefficient of 0.92 for the overall scale [18]. In this study, the internal consistency coefficient (Cronbach α) for the total CSS-12 was calculated as an α of 0.75. The scale consists of 4 dimensions and 12 statements. The scale is a 5-point Likert-type scale (1=never, 2=rarely, 3=sometimes, 4=often, and 5=always).

A total score between 12 and 60 can be obtained from the scale. An increase in the score obtained from the scale is interpreted as an increase in the level of cyberchondria. The total cyberchondria score is calculated by summing the scores obtained from each question.

The CSS-12 consists of 4 dimensions: excessiveness (EXC), which assesses increasing or repeated health-information searching (items 1, 3, and 6); distress (DIST), which assesses anxiety or distress resulting from searching (items 4, 8, and 9); reassurance (REAS), which assesses searching attempts that lead individuals to seek medical support (items 5, 11, and 12); and compulsion (COMP), which assesses searching that may interfere with online or other aspects of daily life (items 2, 7, and 10) [18].

#### IHPS

The Health Perception Scale, used to assess health perception, was developed by Diamond et al [15]. The validity and reliability of the Turkish version were established by Kadıoğlu and Yıldız, who reported a Cronbach α coefficient of 0.77 for the total scale [16]. For this study sample, the internal consistency of the IHPS was found to be an α of 0.69. It is a 5-point Likert-type scale consisting of 15 items and 4 subfactors (control center, self-awareness, certainty, and importance of health). The scale consists of 6 items with positive statements (1, 5, 9, 10, 11, and 14) and 9 items with negative statements (2, 3, 4, 6, 7, 8, 12, 13, and 15). Each item is scored as follows: “1, strongly disagree”; “2, disagree”; “3, undecided”; “4, agree”; and “5, strongly agree.” Negative statements are scored in reverse (5=strongly disagree, 4=disagree, 3=undecided, 2=agree, and 1=strongly agree). The scale ranges from a minimum score of 15 to a maximum score of 75. A high score indicates a high perception of health, while a low score indicates a low perception of health.

Control center (COM) subdimension aims to determine whether the individual attributes being healthy to factors outside themselves (luck, fate, or religious belief), that is, whether they gather the control center in themselves for being healthy and their confidence in themselves to change their health (items 2, 3, 4, 12, and 13).

Self-awareness (SAW) subdimension aims to determine the level of the individual’s belief about whether being healthy is in their own hands regarding their self-awareness perception about exercise and proper nutrition for being healthy (items 5, 10, and 14).

Certainty (CER) subdimension aims to determine whether the individual has a definite idea about what they should do to stay healthy and be healthier (items 6, 7, 8, and 15).

Importance of health (IOH) subdimension aims to determine how much importance the individual gives to their health, to what extent they make financial sacrifices in this regard, and whether the importance they give to health is one of the priorities in their life (items 1, 9, and 11) [16].

#### PWBS-OP

The scale was developed and validated for the Turkish older population by Gümüş Demir, with a reported Cronbach α of 0.89 [17]. In this study, the internal consistency coefficient for the PWBS-OP was determined to be an alpha of 0.92. PWBS-OP consists of 15 items and a 5-point Likert scale, ranging from 1 (strongly disagree) to 5 (strongly agree). No cutoff score has been determined for the scale. The minimum score that can be obtained from the scale is 15, and the maximum score is 75. Items 8 and 13 are scored in reverse. Higher scores indicate a higher level of well-being [17].

### Ethical Considerations

This study was conducted in accordance with the Declaration of Helsinki. The study was approved by the Akdeniz University Medical Scientific Research Ethics Committee (TBAEK-511, dated July 25, 2024). The necessary legal permissions were obtained from the Directorate of the Center for Applied Research on Aging to conduct the research. In addition, informed consent was obtained from all participants who volunteered to participate in this study.

### Statistical Analysis

The study data were analyzed using SPSS 25.0 for Windows (SPSS Inc). For descriptive findings, categorical variables were presented as numbers and percentages, while continuous variables were presented as mean (SD) and minimum to maximum values. The Kolmogorov-Smirnov normality test was used to determine whether the data showed a normal distribution. It was determined that the data, except for the CSS-12 total score, did not conform to normal distribution. In the analysis of nonparametric data, the Mann-Whitney *U* test, Kruskal-Wallis test, and Spearman correlation were used. For variables where the difference was significant, pairwise comparisons between groups were evaluated using the post hoc Dunn test (with Bonferroni correction). Pearson correlation analysis was used in the analysis of parametric data. Factors playing a role in predicting participants’ health perception and psychological well-being levels were evaluated using multiple linear regression analysis. Variables found to be statistically significant in the pairwise comparison were included in the regression model. Categorical variables were included in the regression analysis by defining one dummy variable (coded as 0‐1) representing the number of categories in the variable. Multiple linear regression models were created using the forward selection method. The normality of the residuals obtained from the final regression model was checked using the Kolmogorov-Smirnov test. All the findings were tested at a significance level of 0.05.

## Results

### Characteristics of Participants

The mean age of the 352 participants was 67.4 (SD 4.7, range 60-87) years. Of the 352 participants, 136 (38.6%) were first-year students, 245 (69.6%) were men, 207 (58.8%) were married, and 172 (48.9%) were university graduates or above (Table 1). No statistically significant difference was found between the daily internet usage times of the 4 classes (*P*=.10).

In terms of health status, 229 (65.1%) participants reported living with at least one chronic disease, and 199 (56.5%) spent between 1 and 2 hours on the internet daily. Participants demonstrated a high level of engagement with online health information; 167 (47.4%) participants reported searching the internet before starting a treatment recommended by their physician. While 247 (70.2%) participants were unsure about the reliability of online health resources, official health care organization websites (n=189, 52.8%) and social networks (n=133, 37.8%) were the most preferred sources for health searches. Notably, a small but significant minority (n=15, 4.3%) reported discontinuing medical treatments based on information found online (Table 2).

**Table 1.** Sociodemographic characteristics of Tazelenme University students (N=352).

Characteristics	Values
Age (years), mean (SD)	67.4 (4.7)
Class, n (%)	
Class 1	136 (38.6)
Class 2	78 (22.2)
Class 3	99 (28.1)
Class 4	39 (11.1)
Gender, n (%)	
Female	107 (30.4)
Male	245 (69.6)
Marital status, n (%)	
Married	207 (58.8)
Divorced	70 (19.9)
Widowed	63 (17.9)
Single	12 (3.4)
Education level^a^, n (%)	
Primary school	25 (7.1)
Secondary school	16 (4.5)
High school	139 (39.5)
University or higher	172 (48.9)
Smoking, n (%)	
Nonsmoker	147 (41.8)
Ex-smoker	158 (44.9)
Current smoker	47 (13.4)
Economic status, n (%)	
Income<expenditure	74 (21)
Income=expenditure	218 (61.9)
Income>expenditure	60 (17)
Living arrangement, n (%)	
Spouse	149 (42.3)
Alone	112 (31.8)
Spouse and children	55 (15.6)
Children	30 (8.5)
Relatives	6 (1.7)
Health insurance, n (%)	
Yes	345 (98)
No	7 (2)

a Educational status represents the participants’ formal education level prior to their enrollment at Tazelenme University

**Table 2.** Medical data of participants and their health information search characteristics on the internet (N=352).

Characteristics	Values
Presence of chronic disease, n (%)	
Yes	229 (65.1)
No	123 (34.9)
Number of physician visits in the past year, n (%)	
Never	24 (6.8)
1‐3 times	159 (45.2)
4‐6 times	104 (29.5)
7‐9 times	29 (8.2)
≥10 times	36 (10.2)
Daily internet use duration, n (%)	
1‐2 hours	199 (56.5)
3‐4 hours	114 (32.4)
5‐6 hours	28 (8.0)
>6 hours	11 (3.1)
Unrecommended medical testing in the past year, n (%)	
Yes	117 (33.2)
No	235 (66.8)
Internet-based self-diagnosis and self-medication in the past year, n (%)	
Yes	13 (3.7)
No	339 (96.3)
Belief of having an undiagnosed illness, n (%)	
Yes	47 (13.4)
No	305 (86.6)
Frequency of searching for health topics online, n (%)	
Always	33 (9.4)
Often	52 (14.8)
Sometimes	162 (46.0)
Rarely	85 (24.1)
Never	20 (5.7)
Internet research before starting prescribed treatment, n (%)	
Yes	167 (47.4)
No	185 (52.6)
Internet-driven treatment discontinuation, n (%)	
Yes	15 (4.3)
No	337 (95.7)
Perceived reliability of online health resources, n (%)	
Not reliable	73 (20.7)
Reliable	32 (9.1)
Not sure	247 (70.2)
Internet resources used for health searches^a^^,^^b^, n (%)	
Social networks	133 (37.8)
Blogs	19 (5.4)
Forums	14 (4.0)
Personal websites	63 (17.9)
Health organization websites	186 (52.8)
Newspaper articles, news stories, or academic articles	119 (33.8)

a Based on a multiple-response question, participants could select more than one option.

b Percentages are calculated based on the total number of participants (N=352) and may exceed 100% due to multiple selections.

### Score Averages of All Scales

Descriptive statistics for the assessment tools revealed a mean CSS-12 total score of 28.50 (SD 6.96), indicating the sample’s general level of cyberchondria. The mean scores for health perception (IHPS) and psychological well-being (PWBS-OP) were 53.74 (SD 6.53) and 58.82 (SD 8.41), respectively. Within the subdimensions of the CSS-12, “excessiveness” (mean 8.31, SD 2.72) and “reassurance seeking” (mean 7.57, SD 2.45) were observed to have the highest mean values (Table 3).

**Table 3.** Subscale scores and total score averages of all scales.

Subscales and total scores	Mean (SD)	Minimum to maximum
CSS-12^a^-excessiveness	8.31 (2.72)	3‐15
CSS-12-distress	7.40 (2.39)	3‐15
CSS-12-reassurance	7.57 (2.45)	3‐15
CSS-12-compulsion	5.21 (2.27)	3‐15
CSS-12-total Score	28.50 (6.96)	12‐53
IHPS^b^-COM^c^	18.01 (3.62)	8‐25
IHPS-SAW^d^	11.05 (2.09)	5‐15
IHPS-CER^e^	13.39 (3.11)	4‐20
IHPS-IOH^f^	10.56 (1.94)	4‐15
IHPS-total score	53.74 (6.53)	39‐75
PWBS-OP-total score^g^	58.82 (8.41)	30‐74

a CSS-12: Cyberchondria Severity Scale Short Form.

b IHPS: Individual Health Perception Scale.

c COM: control center.

d SAW: self-awareness.

e CER: certainty.

f IOH: importance of health.

g PWBS-OP: Psychological Well-Being Scale for the Older People.

### The Relationship of Sociodemographic Characteristics, Medical Data, and Internet Usage With IHPS and PWBS-OP

Comparative analyses indicated that health perception levels were significantly higher among male participants (*P*=.02) and those with a high school education or above (*P*=.047; Table 4). Conversely, psychological well-being was found to be lower in participants who were married (*P*=.002), those with lower income levels (*P*<.001), and those who reported thinking they might have an undiagnosed illness (*P*<.001). The psychological well-being level of older people living with their spouse and children was found to be lower than that of those living alone (*P*=.02; Table 4). The analysis demonstrated no statistically significant differences in IHPS and PWBS-OP total scores across classes, smoking, and presence of health insurance, all failing to reach the formal significance threshold (*P*>.05).

According to the pairwise comparisons in the Dunn test with Bonferroni correction displayed, PWBS-OP total scores differed significantly only between married participants (mean rank 162.70, SD 100.96) and widowed or single participants (mean rank 210.20, SD 101.10; adjusted *P*=.002). No statistically significant differences were observed between married and divorced participants (adjusted *P*=.56) or between divorced and widowed or single participants (adjusted *P*=.26). Individuals who are widowed or single tend to have higher total PWBS-OP scores. The statistically significant variance in health perception based on education level identified in the overall Kruskal-Wallis analysis (*P*=.047) was not robust enough to be sustained during pairwise comparisons after applying the Dunn test with Bonferroni correction designed to mitigate type I error. Nonetheless, an examination of the mean ranks indicates that older adults with a primary or middle school education level tended to exhibit lower health perception compared to those with high school (adjusted *P*=.06) and university (adjusted *P*=.06) degrees. Pairwise comparisons in the Dunn test with Bonferroni correction were performed to investigate the specific differences in PWBS-OP total scores across perceived economic status groups. The post hoc analysis revealed that participants who perceived their income as “greater than expenses” (mean rank 232.97, SD 86.05) exhibited statistically significant and substantially higher PWBS-OP total scores compared to both the “income equal to expenses” group (mean rank 170.95, SD 100.08; test statistic=−62.019; *P*<.001) and the “income less than expenses” group (mean rank 147.07, SD 101.72; test statistic=−85.892; *P*<.001). Conversely, the difference in PWBS-OP total scores between the “income equal to expenses” and “income less than expenses” groups was not statistically significant after applying the Dunn test with Bonferroni correction (test statistic=−23.873; *P*=.24). Following the significant overall Kruskal-Wallis test (*P*=.02), pairwise comparisons in the Dunn test with Bonferroni correction were performed to identify the source of variation across living arrangement groups. The post hoc analysis confirmed that participants living alone (mean rank 197.04, SD 98.23) exhibited statistically significant higher PWBS-OP total scores compared to those living with their spouse and children (mean rank 143.92, SD 97.82; test statistic=53.122; adjusted *P*=.009).

**Table 4.** Differences in IHPS^a^ and PWBS-OP^b^ scores according to the sociodemographic characteristics of participants.

Characteristics	IHPS total score		PWBS-OP total score	
	Mean (SD)	*P* value	Mean (SD)	*P* value
Gender		.02^c^		.15^c^
Male	55.0 (5.6)		57.9 (8.4)	
Female	53.2 (6.8)		59.2 (8.4)	
Marital status		.44^d^		.002^d^
Married	53.6 (6.3)		57.7 (8.4)	
Divorced	54.6 (6.6)		59.2 (7.9)	
Widowed or single	53.3 (7.1)		61.6 (8.5)	
Educational status		.047^d^		.05^d^
Primary or secondary school	51.2 (5.6)		60.8 (9.3)	
High school	54.2 (6.8)		58.7 (8.1)	
University or higher	54.0 (6.4)		58.5 (8.4)	
Economic status		.44^d^		<.001^d^
Income<expenditure	52.9 (6.6)		56.2 (8.9)	
Income=expenditure	54.0 (6.6)		58.4 (8.2)	
Income>expenditure	53.7 (6.4)		63.5 (6.5)	
Living arrangement		.18^d^		.02^d^
Spouse	54.4 (6.2)		58.6 (8.1)	
Alone	53.7 (6.9)		60.6 (7.9)	
Spouse and children	52.4 (6.9)		56.5 (8.3)	
Children or relatives	53.3 (6.1)		57.7 (10.2)	

a IHPS: Individual Health Perception Scale.

b PWBS-OP: Psychological Well-Being Scale for the Older People.

c Mann-Whitney *U* test.

d Kruskal-Wallis test.

Participants without chronic illnesses had significantly higher levels of perceived health (*P*=.001). PWBS-OP total score of those with chronic disease was determined to be lower than that of those that of without chronic disease (*P*=.008). The IHPS score average of older people who do not think they have an undiagnosed disease is higher than that of those who have such thoughts (*P*=.02). PWBS-OP total score of those who never went to a physician examination in the past year was found to be higher than that of those who did (*P*<.001). In the Dunn test with Bonferroni correction, older individuals who had not had a physician’s examination in the past year were found to have higher PWBS-OP total scores than other groups. The Dunn test with Bonferroni correction revealed statistically significant differences between those who visited a physician 1 to 3 times and those who visited 7 to 9 times (*P*=.01), between those who visited 7 to 9 times and those who did not visit at all (*P*=.001), between those who visited 10 times and those who did not visit at all (*P*=.02), and between those who did not visit at all and those who visited 4 to 6 times (*P*=.03). PWBS-OP total score of those who had medical tests done without a physician’s recommendation in the past year was determined to be higher than those who did not have tests done (*P*=.002). PWBS-OP total score of those who do not think they have an undiagnosed disease was found to be higher than those who have such thoughts (*P*<.001). PWBS-OP total score of those who stated that they “always” research health-related topics on the internet was found to be higher than the groups giving other answers (*P*=.02). The Dunn test with Bonferroni correction showed that those who always search for health information online had the highest total PWBS-OP score compared to other groups. In pairwise comparisons, statistically significant differences were found between those who frequently search for health information online and those who always do so (*P*=.08) and between those who always do so and those who sometimes do so (*P*=.03; Table 5). The analysis demonstrated no statistically significant differences in IHPS and PWBS-OP total scores across daily internet use duration, internet-based self-diagnosis and self-medication in the past year, internet research before starting prescribed treatment, internet-driven treatment discontinuation, and perceived reliability of online health resources, all failing to reach the formal significance threshold (*P*>.05).

**Table 5.** Comparison of IHPS^a^ and PWBS-OP^b^ scores based on participants’ medical data and internet usage habits.

Characteristics	IHPS total score		PWBS-OP total score	
	Mean (SD)	*P* value	Mean (SD)	*P* value
Presence of chronic disease		.001^c^		.008^c^
Yes	52.9 (6.4)		57.0 (8.3)	
No	55.4 (6.6)		60.4 (8.4)	
Number of physician visits in the past year		.48^d^		<.001^d^
Never	55.3 (7.4)		63.4 (7.8)	
1‐3	53.9 (6.4)		60.1 (7.8)	
4‐6	53.8 (6.9)		57.9 (7.8)	
7‐9	51.8 (5.2)		54.8 (7.7)	
≥10	53.4 (6.6)		55.9 (10.9)	
Unrecommended medical testing in the past year		.31^c^		.002^c^
Yes	54.2 (6.5)		60.8 (7.0)	
No	53.5 (6.5)		57.8 (8.8)	
Belief of having an undiagnosed illness		.02^c^		<.001^c^
Yes	51.4 (5.5)		53.8 (9.5)	
No	54.1 (6.6)		59.6 (7.9)	
Frequency of searching for health topics online		.86^d^		.02^d^
Always	54.7 (9.1)		63.4 (6.6)	
Often	53.6 (6.9)		57.0 (8.1)	
Sometimes	53.4 (5.9)		58.4 (8.3)	
Rarely	53.9 (6.1)		59.4 (7.9)	
Never	54.6 (7.1)		57.2 (11.7)	

a IHPS: Individual Health Perception Scale.

b PWBS-OP: Psychological Well-Being Scale for the Older People.

c Mann-Whitney *U* test.

d Kruskal-Wallis test.

To delineate specific differences across physician visit frequency groups following the general Kruskal-Wallis analysis, pairwise comparisons with Bonferroni correction were conducted. Significant variations were observed among groups. Specifically, participants reporting no physician visits (mean rank 234.71, SD 100.06) exhibited significantly higher PWBS-OP scores compared to those visiting 7 to 9 times (mean rank 123.33, SD 92.66; test statistic=111.381; adjusted *P*=.001), 10 or more times (mean rank 153.40, SD 108.34; test statistic=81.306; adjusted *P*=.02), and 4 to 6 times (mean rank 165.12, SD 95.99; test statistic=69.588; adjusted *P*=.03). Furthermore, participants with 1 to 3 visits (mean rank 190.08, SD 99.74) also demonstrated significantly higher scores than those with 7 to 9 visits (mean rank 123.33, SD 92.66; test statistic=66.757; adjusted *P*=.01). No statistically significant differences were found between the “none” and “1 to 3 times” groups (adjusted *P*=.45) or among the groups with 4 or more visits. Participants reporting that they “always” search for health-related topics on the internet (mean rank 229.71, SD 87.80) exhibited significantly higher PWBS-OP scores compared to those searching “frequently” (mean rank 154.14, SD 101.58; test statistic=75.568; adjusted *P*=.008) and “sometimes” (mean rank 171.25, SD 99.94; test statistic=58.459; adjusted *P*=.03). No statistically significant differences were found among other pairwise comparisons after applying the stringent Bonferroni correction, notably between the “always” group and the “never” (adjusted *P*>.99) or “rarely” (adjusted *P*=.20) groups.

### Correlations Between Participants’ Characteristics and CSS-12, IHPS, and PWBS-OP Scores

In the research, a negative and low-level significant correlation was found between the CSS-12 compulsion subscale and the COM subscale score of IHPS and between participants’ age and the SAW subscale score of IHPS (*P*<.001 and *P*<.05, respectively). A negative and low-level significant correlation was found between CSS-12-distress, CSS-12-compulsion, and CSS-12-total score and the CER subscale score of IHPS (*P*<.001). A positive and low-level significant correlation was found between CSS-12-reassurance and CSS-12-total Score and the IOH subscale score of IHPS (*P*<.05). A positive and low-level significant correlation was found between participants’ age and IHPS total score and PWBS-OP total score (*P*<.05). A negative and low-level significant correlation was found between CSS-12-distress, CSS-12-compulsion, and CSS-12-total score and IHPS total score and PWBS-OP total score (*P*<.05; Table S1 in Multimedia Appendix 1).

### Multiple Linear Regression Analysis of Factors Affecting Health Perception and Psychological Well-Being in Highly Educated Older Adults Engaged in Lifelong Learning

According to the multiple linear regression analysis, the presence of chronic disease was the only significant positive predictor of higher health perception levels (*B*=2.16, 95% CI 1.721-3.593; *P*=.003), whereas factors such as age, gender, education level, and CSS-12 total scores did not demonstrate a statistically significant impact (*P*>.05). Psychological well-being (PWBS-OP) was significantly and positively predicted by age (*B*=0.22, 95% CI 0.037-0.399; *P*=.02), marital status (*B*=1.67, 95% CI 1.064-2.716; *P*=.002), economic status (*B*=2.10, 95% CI 1.729-3.464; *P*=.003), and the thought of having an undiagnosed disease (*B*=3.38, 95% CI 1.038-5.830; *P*=.007), whereas it was significantly and negatively predicted by medical examinations in the past year (*B*=−1.29, 95% CI −2.093 to 0.485; *P*=.002), undergoing examinations without a physician’s recommendation (*B*=−2.04, 95% CI −3.794 to −0.290; *P*=.02), searching for health-related topics on the internet (*B*=−1.26, 95% CI −2.152 to −0.360; *P*=.006), and CSS-12 total scores (*B*=−0.13, 95% CI −0.261 to −0.060; *P*=.04; Table S2 in Multimedia Appendix 1).

## Discussion

### Principal Findings

In this study, increased levels of cyberchondria were found to be associated with significant decreases in both health perception and psychological well-being. The findings demonstrated that age, marital status, economic status, and the absence of undiagnosed illness had significant positive effects on psychological well-being. Particularly, the absence of undiagnosed illness positively influenced the psychological well-being of highly educated older adults engaged in lifelong learning.

Interestingly, this study observed slight but significant increases in health perception and psychological well-being as age advanced. This phenomenon has been explained in the literature by factors such as increased life experience, acceptance, and meaning-seeking among older individuals [19]. However, this finding is not consistent across all studies. For instance, Vuorre and Przybylski [20] reported in their analysis across 168 countries that the association between internet and mobile technology use and well-being did not differ significantly by age and was generally minimal. This situation may vary according to cultural factors, individual coping styles, and levels of digital awareness among highly educated older adults engaged in lifelong learning.

The significantly lower psychological well-being levels found among women compared to men are also noteworthy. Chen et al [21] indicated that older women are psychologically more vulnerable due to higher loneliness, lack of social support, and caregiving responsibilities. Similarly, Li et al [22] revealed that women developed higher anxiety when facing uncertainty in the pandemic context. Older women often serve as primary “health managers” or informal caregivers within family networks. When navigating digital health ecosystems, familial responsibility can manifest as a heavy psychological burden. Unlike men, older women tend to be more meticulous and overly cautious in online symptom checking, driven by a sense of duty to protect both their own well-being and the well-being of those they are responsible for. As web-based health resources generate highly ambiguous medical information, this extensive data searching behavior without formal clinical filtering triggers a cycle of cognitive fatigue and cyberchondria. Consequently, the low psychological well-being in older women may not only be a reflection of structural vulnerability but also a direct result of the overly cautious, digitally driven overanalyzing loop that disproportionately affects highly educated older women engaged in lifelong learning.

The negative relationship observed between worsening economic conditions and psychological well-being reveals that financial security serves as a fundamental buffering mechanism for mental health in older adults [23]. Economic vulnerability not only increases individual stress levels but also damages well-being by limiting social participation and sense of belonging [6]. For highly educated older adults engaged in lifelong learning, quality of life is not just about food and shelter; it is about attending courses, buying books, and participating in intellectual communities. The economic crisis directly undermines this lifestyle and identity. What they are experiencing is not just economic stress, but an identity disruption. The person wants to be educated, knowledgeable, and intellectually active, but their budget does not allow it. This gap (higher education vs lower economic status) creates deep psychological unease in their personality. As educated individuals can read macroeconomic trends, inflation curves, and the future more rationally, they are more susceptible to a wave of anticipatory anxiety about the future.

In individuals with thoughts of undiagnosed illness, lower levels of health perception and psychological well-being were identified. This finding demonstrates that difficulty coping with uncertainty and diagnostic ambiguity can trigger cyberchondria behaviors and anxiety [22,24]. Highly educated older adults engaged in lifelong learning have solved lifelong problems by “reading and researching.” When faced with an undiagnosed symptom, they exhibit the same reflex and turn to the internet. Highly educated older adults without medical training, however, become overwhelmed by the manipulative information pool on the internet. Owing to their high digital literacy, they conduct far deeper searches than the average user. This, instead of reducing uncertainty, leads to cognitive overload. The greatest bulwark of psychological well-being in old age is “autonomy” and “cognitive control.” Consequently, within this specific cohort, the erosion of psychological well-being is not merely a consequence of fearing a physical illness but rather a reflection of a deeper existential crisis: the collapse of their cognitive autonomy and self-efficacy, which are fundamental pillars of successful aging when confronted with an unresolvable digital information maze.

This study found that more frequent physician visits in the past year, undergoing medical tests without physician recommendation in the past year, internet-based health research behavior, and cyberchondria behavior were significantly and negatively associated with the psychological well-being levels of highly educated older adults engaged in lifelong learning. The literature also indicates that excessive and inappropriate searching for health information online was associated with an increased likelihood of health anxiety and decreased psychological well-being in individuals [25]. Particularly in highly educated older adults engaged in lifelong learning, despite increased access to technological information, health anxiety and stress symptoms are associated with cyberchondria [3,7].

Intensive and complex health information online, especially in older adults with high health anxiety, is associated with an increased likelihood of worry and fear, which may be linked to impaired psychological well-being. Additionally, using information obtained from the internet without seeking expert opinion can negatively associate with health perception by causing misdiagnosis and unnecessary tests. As web-based health information is inherently ambiguous and probabilistic, this intensive data consumption without formal clinical filtration intensifies latent health anxiety rather than mitigating it [9]. The unique pathway-based insight offered by this study is that this digitally driven anxiety directly translates into concrete, uncoordinated medical overconsumption. To resolve the cognitive dissonance generated by alarmist online information, these highly educated older adults engaged in lifelong learning bypass professional triage, leading to frequent physician visitations and self-directed, unrecommended diagnostic testing. Every ambiguous test result or clinical nuance, in turn, sparks further online cross-referencing, reinforcing the cyberchondria cycle and severely deteriorating both their psychological well-being and their trust in health care systems [26]. Consequently, our findings emphasize that in highly educated older cohorts, technology use is a double-edged sword; high digital literacy, when uncoupled from the ability to filter out cyberchondria-inducing medical noise, transforms a cognitive asset into a profound source of psychological and systemic vulnerability.

The impact of CSS-12’s “distress” and “compulsion” subdimensions reveals a distinct process-oriented vulnerability in highly educated older adults. While cyberchondria is widely conceptualized as a compulsive, anxiety-driven pattern [24,27], in this specific cohort, it transforms an ingrained problem-solving orientation into a maladaptive loop. Driven to resolve somatic uncertainty, these digitally literate individuals engage in exhaustive online cross-referencing. However, because they possess the cognitive capacity to analyze complex data but lack formal clinical frameworks to filter it, this ambiguous web-based information precipitates severe cognitive overload. Ultimately, this pathway constitutes an active cognitive trap where an older individual’s intellectual resources and drive for autonomy paradoxically undermine their mental resilience, thereby eroding subjective well-being.

The finding that 65% (n=229) of participants managed chronic conditions and underwent multiple examinations highlights a unique process-oriented mechanism within highly educated older adults engaged in lifelong learning. While clinical status traditionally shapes subjective health perception [21,28], a chronic diagnosis in this demographic threatens cognitive autonomy, driving individuals to deploy their digital literacy for intensive online research. However, navigating inherently ambiguous web-based health data creates an overanalysis trap that triggers a compulsive cascade of medical cross-checking and heightened anxiety. This pathway-based dynamic explains why the complete absence of chronic disease history emerged as the strongest positive predictor of health perception. Spared from the somatic triggers that launch them into the exhausting digital maze, these highly educated older adults engaged in lifelong learning avoid cyberchondria-induced cognitive overload, allowing their intellectual resources to function as stable psychological assets rather than anxiety amplifiers.

Particularly in older adults with multiple chronic diseases, deterioration in both physical and psychological functioning is common. Ryff developed the “Psychological Well-being” model, which divides well-being into 6 distinct dimensions: self-acceptance, positive relationships with others, autonomy, environmental mastery, finding purpose and meaning in life, and personal growth [29]. When psychological well-being is evaluated according to the subdimensions of Ryff’s model, cyberchondria behavior can be considered to have negative effects on autonomy and environmental mastery dimensions.

The findings that nearly half (n=167, 47.4%) of the participants engaged in preconsultation internet research and a critical minority (n=15, 4.3%) discontinued medical treatment based on online information expose the behavioral realities of digital health tracking in highly educated older adults engaged in lifelong learning [30]. Evaluating these findings through a process-oriented framework reveals how an older, highly educated demographic’s pursuit of medical autonomy can paradoxically manifest as clinical nonadherence. For these intellectually engaged individuals, researching symptoms prior to a physician visit is an active effort to maintain agency over age-related somatic changes. However, when highly educated individuals cross-reference complex, unverified web data with professional clinical advice, it often generates profound cognitive dissonance. When this dissonance is left unresolved, the pathway-based trajectory shifts from proactive self-care to dangerous behavioral autonomy, driving individuals to independently terminate prescribed treatments. This maladaptive pattern clarifies why an increased frequency of digital health seeking correlates negatively with psychological well-being, as unguided online searches systematically deteriorate uncertainty-coping mechanisms and amplify somatic threat perceptions [6,31]. Furthermore, this distinct nuance reconciles the apparent contradiction with macro-level data, such as Vuorre and Przybylski’s [20] finding that general broadband adoption does not consistently undermine global well-being. The critical insight here is that while general digital connectivity remains benign on a macro scale [20], a targeted, pathway-based exploration reveals that anxiety-driven medical overanalysis by highly educated older adults engaged in lifelong learning presents a direct, acute threat to their psychological resilience and subjective well-being.

Evaluating technology-induced well-being through a population-specific lens reveals a critical process-oriented nuance in our highly educated older cohort: the positive link between cyberchondria’s “reassurance seeking” and health importance reflects an intellectual drive for autonomy rather than pure pathology. This intersection highlights a vital pathway-based pivot; while unguided searches induce anxiety, structured information gathering enhances health self-efficacy [32], converting anxious drives into proactive self-care agency. This adaptive trajectory is mirrored by the participants’ sophisticated information seeking hierarchy, which uniquely included academic literature. However, as established by Cao and Chu [33], the perceived credibility of these mixed-reliability platforms fundamentally dictates subsequent psychological well-being. The resulting cognitive friction between official medical narratives and informal digital commentary ultimately determines whether this information pathway reinforces psychological resilience or precipitates cyberchondria.

This study determined that cyberchondria behavior in highly educated older adults engaged in lifelong learning was associated with an increased likelihood of deterioration in mental and physical health conditions and was negatively associated with psychological well-being. Schuster et al [3] similarly reported that older adults with high health anxiety experienced more stress when researching health information online. Additionally, encountering unreliable information sources provides grounds for older adults to discontinue treatment or self-diagnose [3,30,34]. Thus, for highly educated older adults engaged in lifelong learning, unguided digital health seeking serves as a critical vulnerability that undermines both subjective resilience and objective health safety.

The findings regarding participants’ daily internet usage duration and source preferences in this study underscore the critical importance of enhancing digital health literacy among highly educated older adults engaged in lifelong learning. Recent evidence from Xu and Starcevic [7] highlights that in the modern digital era, health seeking behavior in older adults is intricately linked to mental well-being and health-related quality of life. Improving digital health literacy is now recognized as a vital protective mechanism that shields older individuals from the adverse effects of misinformation while empowering them to make informed and accurate health decisions. Therefore, initiatives aimed at reducing cyberchondria risk must prioritize the development of advanced digital literacy to align with the contemporary technological landscape.

It is emphasized that exposure to health information available online can create difficulties in accessing accurate information and cognitive fatigue, particularly in highly educated older adults engaged in lifelong learning, and therefore, cyberchondria can have negative effects on psychological well-being. Recent research underscores that this cognitive strain fuels cyberchondria, which in turn serves as a significant predictor of decline in various dimensions of Ryff’s psychological well-being model [29], including psychological growth and psychosocial stability [7]. As highly educated older adults engaged in lifelong learning struggle to reconcile conflicting online reports, their “self-acceptance” regarding their health status and “autonomy” in health-related decision-making are compromised, as they become increasingly fused with anxiety-inducing information. Ultimately, this deterioration in eudaimonic well-being dimensions significantly reduces the overall quality of life in this population.

### Limitations and Strengths of the Study

This study has several limitations. First, as the research has a cross-sectional design, establishing causal relationships between variables is limited. Second, the use of an online data collection method, while a methodological convenience that facilitated a larger sample size within the university network, inherently introduces concerns regarding data reliability and selection bias. As our sample consists of highly educated older adults engaged in lifelong learning who are already active internet users, individuals with lower digital access or literacy are not represented in the data. This limits the generalizability of our findings to the broader older population in Turkey. The majority of participants being men (approximately 70%) and having high education levels makes it difficult to generalize the findings to the general older population. Furthermore, the possibility that health care service utilization may have changed following the COVID-19 pandemic was not considered.

The most important strength of the research is addressing the relationship between cyberchondria behavior and psychological well-being in the older population. Thus, digital health seeking behavior and psychological effects in highly educated older adults engaged in lifelong learning, which have not been sufficiently examined in the literature, were determined. By using validated tools such as the CSS-12, this study provides a foundational understanding of digital health seeking behaviors and their psychological impacts on an increasingly tech-savvy older population.

### Conclusion and Recommendations

In conclusion, high levels of cyberchondria behavior in highly educated older adults engaged in lifelong learning negatively associate with psychological well-being levels. Furthermore, while sociodemographic factors may have positive effects on health perception, excessive and uncontrolled internet-based health information seeking can negatively impact the psychological well-being of highly educated older adults engaged in lifelong learning. Accordingly, to reduce the information confusion and misguidance that highly educated older adults engaged in lifelong learning encounter when seeking health information online, it is recommended to expand reliable digital health literacy education, increase internet-based health counseling services by health care professionals, and develop psychological support and awareness programs targeting cyberchondria risk. Additionally, conducting longitudinal research, including older adults from different socioeconomic and cultural levels in future studies, will provide stronger evidence regarding the association between cyberchondria and psychological well-being.

## Supplementary material

10.2196/83371 Multimedia Appendix 1 Additional results.

## Abbreviations

CER: certainty
COM: control center
COMP: compulsion
CSS-12: Cyberchondria Severity Scale Short Form
DIST: distress
EXC: excessiveness
IHPS: Individual Health Perception Scale
IOH: importance of health
PWBS-OP: Psychological Well-Being Scale for Older People
REAS: reassurance
SAW: self-awareness
WHO: World Health Organization
