# Supplementary material for: Factors Associated With Health Perception and Psychological Well-Being Among Third-Age University Students: Cross-Sectional Study on the Role of Cyberchondria
Source: JMIR Hum Factors. 2026 Aug 21;13:e83371. doi: 10.2196/83371 (PMC13496928; doi:10.2196/83371)
Supplement: Supplementary file 1 [file humanfactors-v13-e83371-s001.docx]

# SUPPLEMENTARY MATERIALS A — RESULTS

| **Variables** | **IHPS-COM*** | **IHPS-SAW*** | **IHPS-CER*** | **IHPS-IOH*** | **IHPS-Total Score*** | **PWBS-OP- Total Score*** |
| --- | --- | --- | --- | --- | --- | --- |
| Age* | .048 | **-.122 ^a^** | .065 | .102 | **.113 ^a^** | **.122 ^a^** |
| CSS-12-Excessiveness* | .003 | .037 | -.014 | .041 | .015 | -.037 |
| CSS-12-Distress* | -.091 | -.055 | **-.243 ^b^** | .025 | **-.159 ^b^** | **-.133 ^a^** |
| CSS-12-Reassurance* | -.016 | .001 | -.099 | **.121 ^a^** | -.047 | -.049 |
| CSS-12-Compulsion* | **-.164 ^b^** | .037 | **-.295 ^b^** | .104 | **-.182 ^b^** | **-.117 ^a^** |
| CSS- 12-Total Score** | -.079 | -.002 | **-.225 ^b^** | .**108 ^a^** | **-.121 ^a^** | **-.117 ^a^** |

***Table S1.*** *Correlation between participant age and scale scores* *(n=352)*

**Spearman Correlation, **Pearson Correlation; ^a^p < 0.05, ^b^p < 0.001*

*CSS-12: Cyberchondria severity scale Short Form; IHPS: Individual health perception scale; COM: Control center; SAW: Self-awareness; CER: Certainty; IOH: Importance of health; PWBS-OP: Psychological Well-Being Scale for the Older People*

| **IHPS Total Score** | | | | | | |
| --- | --- | --- | --- | --- | --- | --- |
| **Independent Variables** | Regression coefficient | Standardized regression coefficient (β) | t | p | 95% Confidence Interval | |
| **Age** | 0.12 | 0.08 | 1.54 | 0.124 | -0.032 | 0.262 |
| **Gender** | -1.18 | -0.08 | -1.56 | 0.121 | -2.679 | 0.312 |
| **Education level** | 0.64 | 0.67 | 1.27 | 0.204 | -0.350 | 1.631 |
| **Presence of chronic disease** | 2.16 | 0.16 | 2.95 | **0.003** | 1.721 | 3.593 |
| **Having an undiagnosed disease** | 1.92 | 0.10 | 1.89 | 0.059 | -0.074 | 3.915 |
| **CSS- 12-Total score** | -0.07 | -0.07 | -1.40 | 0.162 | -0.166 | 0.028 |
| **Multiple R = .273 Multiple R^2^ = .075 Durbin-Watson= 2.008 (p= 0.000)** | | | | | | |
| **PWBS-OP Total Score** | | | | | | |
| **Age** | 0.22 | 0.12 | 2.37 | **0.018** | 0.037 | 0.399 |
| **Presence of chronic disease** | 0.93 | 0.05 | 1.03 | 0.305 | -0.853 | 2.716 |
| **Marital status** | 1.67 | 0.18 | 3.18 | **0.002** | 1.064 | 2.716 |
| **Economic status** | 2.10 | 0.15 | 3.02 | **0.003** | 1.729 | 3.464 |
| **The person living together** | -0.74 | -0.09 | -1.58 | 0.114 | -1.660 | 0.180 |
| **Medical examination status in the last 1 year** | -1.29 | -0.16 | -3.15 | **0.002** | -2.093 | 0.485 |
| **Undergoing medical examinations without a doctor's recommendation in the last 1 year** | -2.04 | -0.12 | -2.29 | **0.022** | -3.794 | -0.290 |
| **The thought of having an undiagnosed disease** | 3.38 | 0.14 | 2.72 | **0.007** | 1.038 | 5.830 |
| **Search for health-related topics on the Internet** | -1.26 | -0.15 | -2.76 | **0.006** | -2.152 | -0.360 |
| **CSS- 12 Total score** | -0.13 | -0.11 | -2.06 | **0.040** | -0.261 | -0.060 |
| Multiple R = .459 Multiple R^2^ = .211 Durbin-Watson= 1.838 (p= 0.000) | | | | | | |

***Table S2.*** *Multiple linear regression model analysis with variables affecting health perception levels and psychological well-being of older individuals*

*CSS-12: Cyberchondria severity scale Short Form; IHPS: Individual health perception scale; PWBS-OP: Psychological Well-Being Scale for the Older People*
